# Supplementary material for: End Sequence Analysis Toolkit (ESAT) expands the extractable information from single-cell RNA-seq data
Source: Genome Res. 2016 Oct;26(10):1397–410. doi: 10.1101/gr.207902.116 (PMC5052061; doi:10.1101/gr.207902.116)
Supplement: Supplemental Material [file supp_gr.207902.116_Supp_Materials.tar.gz › ESAT-esat_v0.1/doc/html/classbroad_1_1pda_1_1gene_1_1_gene_with_isoforms.html]

ESAT: broad.pda.gene.GeneWithIsoforms Class Reference


ESAT


- Main Page
- Related Pages
- Packages
- Classes
- Files

- Class List
- Class Index
- Class Hierarchy
- Class Members

All Classes Namespaces Files Functions Variables Enumerator Pages

- broad
- pda
- gene
- GeneWithIsoforms

Public Member Functions |
List of all members

broad.pda.gene.GeneWithIsoforms Class Reference

Inheritance diagram for broad.pda.gene.GeneWithIsoforms:

[legend]

Collaboration diagram for broad.pda.gene.GeneWithIsoforms:

[legend]

|  |  |
| --- | --- |
| Public Member Functions | |
|  | GeneWithIsoforms (Collection<?extends LightweightGenomicAnnotation > exons) |
|  | |
|  | GeneWithIsoforms (LightweightGenomicAnnotation align) |
|  | |
|  | GeneWithIsoforms (String pslString) |
|  | |
|  | GeneWithIsoforms (String chrString, int start, int end) |
|  | |
|  | GeneWithIsoforms (String chr, int start, int end, String name, Strand orientation, Collection< Annotation > exons) |
|  | |
|  | GeneWithIsoforms (String chr, int start, int end, String name, String orientation, List< Integer > exonsStart, List< Integer > exonsEnd) |
|  | |
|  | GeneWithIsoforms (String chr, int start, int end, String name, String orientation, List< Integer > exonsStart, List< Integer > exonsEnd, int blockStart, int blockEnd) |
|  | |
|  | GeneWithIsoforms (String chr, int start, int end, String name, String orientation, List< Integer > exonsStart, List< Integer > exonsEnd, int blockStart, int blockEnd, String[] extraData) |
|  | |
|  | GeneWithIsoforms (String chr, int start, int end, String name, String orientation, List< Integer > exonsStart, List< Integer > exonsEnd, String[] extraData) |
|  | |
|  | GeneWithIsoforms (Gene gene) |
|  | |
| boolean | IsExactOverlappingIsoform (Gene gene) |
|  | |
| boolean | AddIsoform (Gene gene) |
|  | |
| boolean | AddIsoform (Gene gene, boolean updateMainIsoform) |
|  | |
| boolean | addContainedIsoform (Gene isoform) |
|  | |
| boolean | AddAllIsoforms (GeneWithIsoforms overlapper) |
|  | |
| boolean | AddAllIsoforms (GeneWithIsoforms overlapper, boolean updateMainIsoform) |
|  | |
| boolean | AddAllIsoNotIncludedforms (GeneWithIsoforms other) |
|  | |
| Collection< Gene > | getAllIsoforms () |
|  | |
| Collection< Gene > | getAllIsoforms (boolean includeContainingIsoform) |
|  | |
| void | setBedScore (double scr) |
|  | |
| void | setExtraFields (double[] scores) |
|  | |
| void | updateScrToBedScore (double score) |
|  | |
| List< Annotation > | getScoredExons () |
|  | |
| boolean | overlapsByGenomicRegion (IntervalTree< GeneWithIsoforms > otherTree, boolean considerOrientation) |
|  | |
| void | dedup () |
|  | |
| Gene | findCompatibleGenes (IntervalTree< GeneWithIsoforms > overlapTree, int[] numIntrons) |
|  | |
| void | expandUtrs (Integer utr1, Integer utr2) |
|  | |
| void | addSuffixToName (String refName) |
|  | |
| boolean | overlapsExon (LightweightGenomicAnnotation exon) |
|  | |
| boolean | overlapsExon (Gene gene) |
|  | |
| boolean | overlaps (Gene other) |
|  | |
| Gene | getMerged () |
|  | |
| void | cleanIsoforms () |
|  | |
| Collection< Annotation > | getAllIsoformExonSet () |
|  | |
| Collection< Annotation > | getAllIsoformIntronSet () |
|  | |
| Gene | constituentIsoform () |
|  | |
| Gene | constituentIntrons () |
|  | |
| void | colapse (int exonFudgeFactor) |
|  | |
| boolean | almostContains (Gene other, int exonFudgeFactor) |
|  | |
| boolean | almostContains (GeneWithIsoforms other, int exonFudgeFactor) |
|  | |
| boolean | hasOverlapping3PUTR (GeneWithIsoforms other) |
|  | |
| boolean | hasOverlapping5PUTR (GeneWithIsoforms other) |
|  | |
| String | allIsoformsToBED () |
|  | |
| void | standardizeOreintation () |
|  | |
| String | toString (String name) |
|  | |
| Integer | getNumberOfIsoforms () |
|  | |
| Collection<?extends Gene > | selectRandIsoSubset (Integer maxIsoPerLoci) |
|  | |

## Constructor & Destructor Documentation

|  |  |  |  |  |  |
| --- | --- | --- | --- | --- | --- |
| broad.pda.gene.GeneWithIsoforms.GeneWithIsoforms | ( | Collection<?extends LightweightGenomicAnnotation > | *exons* | ) |  |

|  |  |  |  |  |  |
| --- | --- | --- | --- | --- | --- |
| broad.pda.gene.GeneWithIsoforms.GeneWithIsoforms | ( | LightweightGenomicAnnotation | *align* | ) |  |

|  |  |  |  |  |  |
| --- | --- | --- | --- | --- | --- |
| broad.pda.gene.GeneWithIsoforms.GeneWithIsoforms | ( | String | *pslString* | ) |  |

|  |  |  |  |
| --- | --- | --- | --- |
| broad.pda.gene.GeneWithIsoforms.GeneWithIsoforms | ( | String | *chrString*, |
|  |  | int | *start*, |
|  |  | int | *end* |
|  | ) |  |  |

|  |  |  |  |
| --- | --- | --- | --- |
| broad.pda.gene.GeneWithIsoforms.GeneWithIsoforms | ( | String | *chr*, |
|  |  | int | *start*, |
|  |  | int | *end*, |
|  |  | String | *name*, |
|  |  | Strand | *orientation*, |
|  |  | Collection< Annotation > | *exons* |
|  | ) |  |  |

|  |  |  |  |
| --- | --- | --- | --- |
| broad.pda.gene.GeneWithIsoforms.GeneWithIsoforms | ( | String | *chr*, |
|  |  | int | *start*, |
|  |  | int | *end*, |
|  |  | String | *name*, |
|  |  | String | *orientation*, |
|  |  | List< Integer > | *exonsStart*, |
|  |  | List< Integer > | *exonsEnd* |
|  | ) |  |  |

|  |  |  |  |
| --- | --- | --- | --- |
| broad.pda.gene.GeneWithIsoforms.GeneWithIsoforms | ( | String | *chr*, |
|  |  | int | *start*, |
|  |  | int | *end*, |
|  |  | String | *name*, |
|  |  | String | *orientation*, |
|  |  | List< Integer > | *exonsStart*, |
|  |  | List< Integer > | *exonsEnd*, |
|  |  | int | *blockStart*, |
|  |  | int | *blockEnd* |
|  | ) |  |  |

|  |  |  |  |
| --- | --- | --- | --- |
| broad.pda.gene.GeneWithIsoforms.GeneWithIsoforms | ( | String | *chr*, |
|  |  | int | *start*, |
|  |  | int | *end*, |
|  |  | String | *name*, |
|  |  | String | *orientation*, |
|  |  | List< Integer > | *exonsStart*, |
|  |  | List< Integer > | *exonsEnd*, |
|  |  | int | *blockStart*, |
|  |  | int | *blockEnd*, |
|  |  | String[] | *extraData* |
|  | ) |  |  |

|  |  |  |  |
| --- | --- | --- | --- |
| broad.pda.gene.GeneWithIsoforms.GeneWithIsoforms | ( | String | *chr*, |
|  |  | int | *start*, |
|  |  | int | *end*, |
|  |  | String | *name*, |
|  |  | String | *orientation*, |
|  |  | List< Integer > | *exonsStart*, |
|  |  | List< Integer > | *exonsEnd*, |
|  |  | String[] | *extraData* |
|  | ) |  |  |

|  |  |  |  |  |  |
| --- | --- | --- | --- | --- | --- |
| broad.pda.gene.GeneWithIsoforms.GeneWithIsoforms | ( | Gene | *gene* | ) |  |

## Member Function Documentation

|  |  |  |  |  |  |
| --- | --- | --- | --- | --- | --- |
| boolean broad.pda.gene.GeneWithIsoforms.AddAllIsoforms | ( | GeneWithIsoforms | *overlapper* | ) |  |

|  |  |  |  |
| --- | --- | --- | --- |
| boolean broad.pda.gene.GeneWithIsoforms.AddAllIsoforms | ( | GeneWithIsoforms | *overlapper*, |
|  |  | boolean | *updateMainIsoform* |
|  | ) |  |  |

|  |  |  |  |  |  |
| --- | --- | --- | --- | --- | --- |
| boolean broad.pda.gene.GeneWithIsoforms.AddAllIsoNotIncludedforms | ( | GeneWithIsoforms | *other* | ) |  |

Here is the call graph for this function:

|  |  |  |  |  |  |
| --- | --- | --- | --- | --- | --- |
| boolean broad.pda.gene.GeneWithIsoforms.addContainedIsoform | ( | Gene | *isoform* | ) |  |

Here is the call graph for this function:

|  |  |  |  |  |  |
| --- | --- | --- | --- | --- | --- |
| boolean broad.pda.gene.GeneWithIsoforms.AddIsoform | ( | Gene | *gene* | ) |  |

|  |  |  |  |
| --- | --- | --- | --- |
| boolean broad.pda.gene.GeneWithIsoforms.AddIsoform | ( | Gene | *gene*, |
|  |  | boolean | *updateMainIsoform* |
|  | ) |  |  |

Here is the call graph for this function:

|  |  |  |  |  |  |
| --- | --- | --- | --- | --- | --- |
| void broad.pda.gene.GeneWithIsoforms.addSuffixToName | ( | String | *refName* | ) |  |

Here is the caller graph for this function:

|  |  |  |  |  |
| --- | --- | --- | --- | --- |
| String broad.pda.gene.GeneWithIsoforms.allIsoformsToBED | ( |  | ) |  |

Here is the call graph for this function:

|  |  |  |  |
| --- | --- | --- | --- |
| boolean broad.pda.gene.GeneWithIsoforms.almostContains | ( | Gene | *other*, |
|  |  | int | *exonFudgeFactor* |
|  | ) |  |  |

Here is the call graph for this function:

Here is the caller graph for this function:

|  |  |  |  |
| --- | --- | --- | --- |
| boolean broad.pda.gene.GeneWithIsoforms.almostContains | ( | GeneWithIsoforms | *other*, |
|  |  | int | *exonFudgeFactor* |
|  | ) |  |  |

Here is the call graph for this function:

|  |  |  |  |  |
| --- | --- | --- | --- | --- |
| void broad.pda.gene.GeneWithIsoforms.cleanIsoforms | ( |  | ) |  |

|  |  |  |  |  |  |
| --- | --- | --- | --- | --- | --- |
| void broad.pda.gene.GeneWithIsoforms.colapse | ( | int | *exonFudgeFactor* | ) |  |

|  |  |  |  |  |
| --- | --- | --- | --- | --- |
| Gene broad.pda.gene.GeneWithIsoforms.constituentIntrons | ( |  | ) |  |

Constructs an artificial gene where the constituent introns (regions that are intronic in all isoforms) are the exons of the gene.

Returns

Here is the call graph for this function:

|  |  |  |  |  |
| --- | --- | --- | --- | --- |
| Gene broad.pda.gene.GeneWithIsoforms.constituentIsoform | ( |  | ) |  |

Here is the call graph for this function:

|  |  |  |  |  |
| --- | --- | --- | --- | --- |
| void broad.pda.gene.GeneWithIsoforms.dedup | ( |  | ) |  |

|  |  |  |  |
| --- | --- | --- | --- |
| void broad.pda.gene.GeneWithIsoforms.expandUtrs | ( | Integer | *utr1*, |
|  |  | Integer | *utr2* |
|  | ) |  |  |

|  |  |  |  |
| --- | --- | --- | --- |
| Gene broad.pda.gene.GeneWithIsoforms.findCompatibleGenes | ( | IntervalTree< GeneWithIsoforms > | *overlapTree*, |
|  |  | int[] | *numIntrons* |
|  | ) |  |  |

Here is the call graph for this function:

|  |  |  |  |  |
| --- | --- | --- | --- | --- |
| Collection<Annotation> broad.pda.gene.GeneWithIsoforms.getAllIsoformExonSet | ( |  | ) |  |

Here is the caller graph for this function:

|  |  |  |  |  |
| --- | --- | --- | --- | --- |
| Collection<Annotation> broad.pda.gene.GeneWithIsoforms.getAllIsoformIntronSet | ( |  | ) |  |

Here is the caller graph for this function:

|  |  |  |  |  |
| --- | --- | --- | --- | --- |
| Collection<Gene> broad.pda.gene.GeneWithIsoforms.getAllIsoforms | ( |  | ) |  |

Here is the caller graph for this function:

|  |  |  |  |  |  |
| --- | --- | --- | --- | --- | --- |
| Collection<Gene> broad.pda.gene.GeneWithIsoforms.getAllIsoforms | ( | boolean | *includeContainingIsoform* | ) |  |

In cases were the containg gene is artificial, like when building gene loci and adding all isoforms contain in the loci. The containing gene is not desired. This methods allows the caller to exclude the containing isoform from the list.

Parameters
:   |  |  |
    | --- | --- |
    | includeContainingIsoform | - boolean flag indicating whether or not to include the containing isoform |

Returns
:   Collection<RefSeqGene> of all the isoforms for this gene

|  |  |  |  |  |
| --- | --- | --- | --- | --- |
| Gene broad.pda.gene.GeneWithIsoforms.getMerged | ( |  | ) |  |

Here is the caller graph for this function:

|  |  |  |  |  |
| --- | --- | --- | --- | --- |
| Integer broad.pda.gene.GeneWithIsoforms.getNumberOfIsoforms | ( |  | ) |  |

|  |  |  |  |  |
| --- | --- | --- | --- | --- |
| List<Annotation> broad.pda.gene.GeneWithIsoforms.getScoredExons | ( |  | ) |  |

Here is the call graph for this function:

|  |  |  |  |  |  |
| --- | --- | --- | --- | --- | --- |
| boolean broad.pda.gene.GeneWithIsoforms.hasOverlapping3PUTR | ( | GeneWithIsoforms | *other* | ) |  |

Here is the call graph for this function:

|  |  |  |  |  |  |
| --- | --- | --- | --- | --- | --- |
| boolean broad.pda.gene.GeneWithIsoforms.hasOverlapping5PUTR | ( | GeneWithIsoforms | *other* | ) |  |

|  |  |  |  |  |  |
| --- | --- | --- | --- | --- | --- |
| boolean broad.pda.gene.GeneWithIsoforms.IsExactOverlappingIsoform | ( | Gene | *gene* | ) |  |

Here is the caller graph for this function:

|  |  |  |  |  |  |
| --- | --- | --- | --- | --- | --- |
| boolean broad.pda.gene.GeneWithIsoforms.overlaps | ( | Gene | *other* | ) |  |

Here is the caller graph for this function:

|  |  |  |  |
| --- | --- | --- | --- |
| boolean broad.pda.gene.GeneWithIsoforms.overlapsByGenomicRegion | ( | IntervalTree< GeneWithIsoforms > | *otherTree*, |
|  |  | boolean | *considerOrientation* |
|  | ) |  |  |

Here is the caller graph for this function:

|  |  |  |  |  |  |
| --- | --- | --- | --- | --- | --- |
| boolean broad.pda.gene.GeneWithIsoforms.overlapsExon | ( | LightweightGenomicAnnotation | *exon* | ) |  |

Here is the call graph for this function:

Here is the caller graph for this function:

|  |  |  |  |  |  |
| --- | --- | --- | --- | --- | --- |
| boolean broad.pda.gene.GeneWithIsoforms.overlapsExon | ( | Gene | *gene* | ) |  |

Here is the call graph for this function:

|  |  |  |  |  |  |
| --- | --- | --- | --- | --- | --- |
| Collection<? extends Gene> broad.pda.gene.GeneWithIsoforms.selectRandIsoSubset | ( | Integer | *maxIsoPerLoci* | ) |  |

Here is the call graph for this function:

|  |  |  |  |  |  |
| --- | --- | --- | --- | --- | --- |
| void broad.pda.gene.GeneWithIsoforms.setBedScore | ( | double | *scr* | ) |  |

|  |  |  |  |  |  |
| --- | --- | --- | --- | --- | --- |
| void broad.pda.gene.GeneWithIsoforms.setExtraFields | ( | double[] | *scores* | ) |  |

Commented out by skadri on 04/02/12 Replaces extra fields for all children of a gene.

|  |  |  |  |  |
| --- | --- | --- | --- | --- |
| void broad.pda.gene.GeneWithIsoforms.standardizeOreintation | ( |  | ) |  |

Here is the call graph for this function:

|  |  |  |  |  |  |
| --- | --- | --- | --- | --- | --- |
| String broad.pda.gene.GeneWithIsoforms.toString | ( | String | *name* | ) |  |

|  |  |  |  |  |  |
| --- | --- | --- | --- | --- | --- |
| void broad.pda.gene.GeneWithIsoforms.updateScrToBedScore | ( | double | *score* | ) |  |

---

The documentation for this class was generated from the following file:

- java/broad/pda/gene/GeneWithIsoforms.java


---

Generated on Fri Aug 15 2014 15:11:00 for ESAT by  

 1.8.7
